# Editorial: Nutraceuticals modulation for oxidative stress in disease and health

**DOI:** 10.3389/fphar.2023.1199293

**Published:** 2023-04-14

**Authors:** Kok-Yong Chin, Ahmad Nazrun Shuid, Siti Fatimah Ibrahim

**Affiliations:** ^1^ Department of Pharmacology, Faculty of Medicine, Universiti Kebangsaan Malaysia, Cheras, Malaysia; ^2^ Department of Pharmacology, Faculty of Medicine, Universiti Teknologi MARA, Sungai Buloh, Malaysia; ^3^ Department of Physiology, Faculty of Medicine, Universiti Kebangsaan Malaysia, Cheras, Malaysia

**Keywords:** antioxidants, free radicals, natural products, reactive oxygen species, redox status

Oxygen is an essential component of life because it is used in aerobic respiration to generate energy from glucose. Reactive oxygen species (ROS) are produced as by-products of cellular respiration. The enzymatic and non-enzymatic antioxidants in the cells maintain the ROS at low levels, whereby they play a physiological role in cellular signalling, differentiation, autophagy and metabolic adaptation (Sena and Chandel, 2012). When the cellular antioxidant capacity is overwhelmed by ROS, a phenomenon known as oxidative stress occurs. The excess ROS could damage macromolecules important to sustain life, such as lipids, nucleic acids and protein (Caliri et al., 2021). The accumulated damage can lead to cellular senescence or dysfunction, causing organ damage and various degenerative diseases and even cancers (Liguori et al., 2018). Oxidative stress can be triggered by many factors in the modern environment, including pollutants, radiation, smoking, alcohol, psychosocial stress, and unhealthy diets (Birch‐Machin and Bowman, 2016; Kerahrodi and Michal, 2020).

Since complete avoidance of factors triggering oxidative stress is not feasible, enhancing the cellular antioxidant system could be an alternative to tackle this problem. Consumption of natural products such as fruits and vegetables has been associated with various health-beneficial effects (Angelino et al., 2019; Wang et al., 2021). Natural products are rich in hydrophilic (such as flavonoids, lignans, phenolic acids, and stilbenes) and lipophilic antioxidants (such as carotenoids and tocochromanols) (Xu et al., 2017), which possess health-enhancing effects. These natural antioxidants can scavenge free radicals, terminate the lipid peroxidation chain, and modulate the cellular antioxidant response. They are also used in combination as in the case of traditional Chinese and other folk medicines to enhance the health effects. Nutraceuticals, which are substances derived from natural products used to prevent or manage chronic diseases, have been receiving attention (Nasri et al., 2014).

In response to the increasing evidence of nutraceuticals in modulating oxidative stress in health and diseases, this Research Topic gathers high-quality papers on the topic. The researchers adopted several approaches to tackle the issue, i.e., using single bioactive compounds or crude extract, traditional Chinese medicine formulation or reviews of previous literature.

Herbs used in traditional medicine are major sources of nutraceuticals, and a topic of active investigation. Using menthol derived from peppermint, Matouk et al. reported attenuation of histopathological and enzymatic changes in the liver caused by sepsis with treatment. These beneficial effects were mediated by the anti-inflammatory, antioxidant and anti-apoptotic properties of menthol. Using the aqueous extract of *Labisia Pumila* (Blume) Fern.-Vill. Var. Alata, Ibrahim et al. reported that the extract promoted the healing of burn wounds. The effects of leaf extract were more potent because it promoted better histomorphometric features and hydroxyproline production in the wound. Nurul Akmal et al. found that methanolic extract of *Piper sarmentosum* prevented lesions and the increase of inflammation and oxidative stress of gastric mucosa in rats subjected to water-immersion restrain stress. Mumtazah Razak et al. compared the antioxidant potential of subcritical water extract of soil and soilless-grown gingers. They found that soil-grown variety yielded higher amounts of extract and antioxidant activities *ex vivo*. In rats, subcritical water extract of soil-grown ginger suppressed inflammation and the product of oxidative stress, while increasing the activity of catalase. Fungi are a major source of bioactive compounds with antioxidant activities. Tang et al. demonstrated that the extract of *Penicillium oxalicum* isolated from *Ligusticum chuanxiong* Hort protected *Caenorhabditis elegans* from thermal, UV and oxidative stress by upregulating antioxidant enzyme and heat-shock protein expression. The extract could also protect against DNA scission in plasmid and lymphocytes.

Two studies demonstrated the antioxidant potential of traditional Chinese medicine formulation in managing health. Lee et al. found that the spleen-tonifying formula, KI Essence extract, promoted the survival of neurons and neurite growth through antioxidant effects and ERK phosphorylation *in vitro*. In animals, the formulation prevented hypomyelination, oxidative stress and inflammation in the brain of maternal immune activation offspring, probably through modulation of gut microbiota. Sun et al. reported that topical application of *Scutellaria baicalensis* Georgi (SBG) improved the skin condition, collagen production and redox status. Through *in vitro* studies, they showed that SBG antagonises REV-ERBα to upregulate BMAL1, thus achieving protection against skin ageing.

The Research Topic also includes three reviews that summarise the health effects of phytoestrogens, herbs and traditional Chinese medicines with antioxidant effects. Jayusman et al. reviewed the potential of phytoestrogens in preserving bone and periodontal health derived from preclinical studies. Although the evidence on periodontal bone loss is limited, they concluded that phytoestrogens could effectively prevent bone resorption and enhance bone formation, thus preventing osteoporosis and alveolar bone loss. In a systematic review, Othman et al. reported that *P. sarmentosum* exerted beneficial effects against diabetes and hypertension from preclinical studies. Meta-analysis of the hypertensive effects showed that *P. sarmentosum* extract induced substantial reduction in systolic, diastolic and mean arterial pressure *in vivo* models. The review of Huang et al. summarized the effects of several traditional Chinese medicine components and formulations with antioxidant, anti-inflammatory, neuroprotective properties, which could potentially be used to manage spinal cord injury with an underlying oxidative stress pathology.

Overall, nutraceuticals hold great promise in preventing and managing chronic diseases through their antioxidant properties. They could complement lifestyle interventions to prevent the progression of the diseases to the stage wherein standard pharmaceuticals are required. However, to adopt them in a clinical setting, more safety and efficacy data in properly planned randomised controlled trials in humans would be required. Standardising the extraction and formulation is also critical in ensuring the effectiveness of the nutraceuticals.
